# Whole-Genome Sequencing of Extended-Spectrum Beta-Lactamase-Producing *Escherichia coli* From Human Infections in Finland Revealed Isolates Belonging to Internationally Successful ST131-C1-M27 Subclade but Distinct From Non-human Sources

**DOI:** 10.3389/fmicb.2021.789280

**Published:** 2022-01-04

**Authors:** Paula Kurittu, Banafsheh Khakipoor, Jari Jalava, Jari Karhukorpi, Annamari Heikinheimo

**Affiliations:** ^1^Department of Food Hygiene and Environmental Health, Faculty of Veterinary Medicine, University of Helsinki, Helsinki, Finland; ^2^Finnish Institute for Health and Welfare, Helsinki, Finland; ^3^Eastern Finland Laboratory Centre Joint Authority Enterprise (ISLAB), Joensuu, Finland; ^4^Finnish Food Authority, Seinäjoki, Finland

**Keywords:** antimicrobial resistance, whole genome sequencing, extended-spectrum beta-lactamases, multidrug resistance, one health

## Abstract

Antimicrobial resistance (AMR) is a growing concern in public health, particularly for the clinically relevant extended-spectrum beta-lactamase (ESBL) and AmpC-producing Enterobacteriaceae. Studies describing ESBL-producing *Escherichia coli* clinical samples from Finland to the genomic level and investigation of possible zoonotic transmission routes are scarce. This study characterizes ESBL-producing *E. coli* from clinical samples in Finland using whole genome sequencing (WGS). Comparison is made between animal, food, and environmental sources in Finland to gain insight into potential zoonotic transmission routes and to recognize successful AMR genes, bacterial sequence types (STs), and plasmids. ESBL-producing *E. coli* isolates (*n* = 30) obtained from the Eastern Finland healthcare district between 2018 and 2020 underwent WGS and were compared to sequences from non-human and healthy human sources (*n* = 67) isolated in Finland between 2012 and 2018. A majority of the clinical isolates belonged to ST131 (*n* = 21; 70%), of which 19 represented O25:H4 and *fimH*30 allele, and 2 O16:H5 and *fimH*41 allele. Multidrug resistance was common, and the most common *bla* gene identified was *bla*_*CTX–M–27*_ (*n* = 14; 47%) followed by *bla*_*CTX–M–15*_ (*n* = 10; 33%). *bla*_*CTX–M–27*_ was identified in 13 out of 21 isolates representing ST131, with 12 isolates belonging to a recently discovered international *E. coli* ST131 C1-M27 subclade. Isolates were found to be genetically distinct from non-human sources with core genome multilocus sequence typing based analysis. Most isolates (*n* = 26; 87%) possessed multiple replicons, with IncF family plasmids appearing in 27 (90%) and IncI1 in 5 (17%) isolates. IncF[F1:A2:B20] replicon was identified in 11, and IncF[F-:A2:B20] in 4 isolates. The results indicate the ST131-C1-M27 clade gaining prevalence in Europe and provide further evidence of the concerning spread of this globally successful pathogenic clonal group. This study is the first to describe ESBL-producing *E. coli* in human infections with WGS in Finland and provides important information on global level of the spread of ESBL-producing *E. coli* belonging to the C1-M27 subclade. The results will help guide public health actions and guide future research.

## Introduction

Antimicrobial resistance (AMR) is an increasing public health concern worldwide. Especially extended-spectrum beta-lactamase (ESBL) and AmpC-producing Enterobacteriaceae have spread globally, and infections caused by resistant bacteria are associated with prolonged hospital stays, increased mortality, and healthcare costs (Ray et al., 2018). Clinically relevant *Escherichia coli* and *Klebsiella pneumoniae* have also become common in community-acquired infections in recent years (Devi et al., 2020). The success of these pathogens is highly attributable to epidemic plasmids, which enable AMR spread *via* horizontal gene transfer (Mathers et al., 2015; Rozwandowicz et al., 2018). Transmission of ESBL-producing *E. coli* from animal, food, and environmental sources have previously been found to account for a limited amount of human ESBL-carriage in selected countries (Börjesson et al., 2016; Mughini-Gras et al., 2019), but gaps in knowledge regarding the wider epidemiology of these bacteria and the role in human carriage and infections still exist. Whole genome sequencing (WGS) allows for in-depth analysis of possible genetic links between different sources, and the role of plasmids in the spread of AMR.

Extraintestinal pathogenic *E. coli* (ExPEC) of sequence type (ST) 131 is a globally spread clonal lineage often associated with multidrug resistance (MDR), conferring resistance to ESBLs and fluoroquinolones (Nicolas-Chanoine et al., 2014). A recently recognized subclade within the dominant ST131 clade C, termed C1-M27, has emerged as a common cause of infection globally (Matsumura et al., 2016; Decano and Downing, 2019) but the occurrence of this subclade in Finland remains unknown.

Carbapenemase-producing *E. coli* isolates of human origin have been described using WGS in Finland previously (Räisänen et al., 2020), but to the best of our knowledge, publications covering ESBL-producing *E. coli* isolates from Finland of human clinical origin to a genomic level are scarce. WGS studies on healthy individuals have been conducted in Finland (Verkola et al., 2019; Gröndahl-Yli-Hannuksela et al., 2020) and a national surveillance program routinely implements only phenotypic characterization for ESBL-producers (Räisänen et al., 2019). The proportion of ESBL-producing *E. coli* in blood and urine specimens in patients in Finland has been relatively low, but steadily increasing during recent years (Räisänen et al., 2019). The national surveillance has found 7.3% of all blood specimens and 3.1 and 7.2% of urine specimens in women and men, respectively, positive for ESBL-producing *E. coli* in 2019. A study investigating fecal samples from healthy, adult volunteers in Finland found the prevalence of ESBL-producing *E. coli* or *K. pneumoniae* to be 6.3% (Gröndahl-Yli-Hannuksela et al., 2020). A gap in knowledge regarding *bla* genes and further genomic characterization of ESBL-producing *E. coli* isolates involved in clinical cases in Finland exists.

This study aimed to characterize ESBL-producing *E. coli* isolates obtained from clinical specimens in Finland. This involves genetic comparisons between previously sequenced isolates from healthy human, animal, food, and environmental sources in Finland to evaluate possible genetic overlap and provide genome-level information of the ESBL-producing *E. coli* found in Finland.

## Materials and Methods

### Collection of Extended-Spectrum Beta-Lactamase-Producing *Escherichia coli* Isolates From Clinical Samples

Altogether 30 ESBL-producing *E. coli* strains were obtained retrospectively from the Eastern Finland Laboratory Centre Joint Authority Enterprise (ISLAB), covering the healthcare district in Eastern Finland. The samples were part of routine practice collected during 2018–2020 from clinical samples, each originating from a different patient. Stored samples were recultivated in March 2020 and transported to the University of Helsinki for further studies with Copan M40 Transystem sterile transport swabs (Copan Transystem, Copan Diagnostics, Italy).

Briefly, antimicrobial susceptibility testing for specimens other than blood and urine was performed using disk diffusion method according to European Committee on Antimicrobial Susceptibility Testing (EUCAST) standards with third-generation cephalosporins (cefpodoxime, ceftazidime, and ceftriaxone), together with amoxicillin-clavulanic acid (Oxoid, Basingstoke, Hampshire, United Kingdom). Specimens other than blood and urine consisted of samples originating from various body sites and tissue types, including joint, scrotum, maxillary sinus, eye conjunctiva, wound, bile, abscess, bronchoalveolar lavage, and abdominal cavity. Additionally, combination disk method (cefotaxime 30 μg and cefotaxime 30 μg + clavulanic acid 10 μg; ceftazidime 30 μg and ceftazidime 30 μg + clavulanic acid 10 μg) and AmpC disk test (Mast Group Ltd., Bootle, United Kingdom) were used for presumptive ESBL-producing *E. coli* isolates. Susceptibility testing for urine samples was performed with a Vitek 2 AST-N385 card (bioMeriéux, Marcy-L’Etoile, France), and for blood samples with both Vitek 2 and disk diffusion method according to EUCAST standards, to ensure rapid and accurate diagnosis in possible septicaemia cases.

### DNA Extraction and Sequencing

Bacterial DNA was extracted and purified with a PureLink Genomic DNA Mini Kit (Invitrogen by Thermo Fischer Scientific, Carlsbad, CA, United States) according to manufacturer’s instructions. The assessment of DNA quality was carried out using a NanoDrop ND-1000 spectrophotometer (Thermo Fischer Scientific, Wilmington, DE, United States) and DNA quantity was measured using a Qubit 2.0 fluorometer (Invitrogen, Life Technologies, Carlsbad, CA, United States). An optical density of 1.8–2.0 at 260/280 nm and a concentration of ≥10 ng/μl with a minimum amount of 0.2 μg were set as thresholds. Library preparation was performed with NEBNext Ultra DNA Library Prep Kit for Illumina (Cat No. E7370L). Sequencing was performed with an Illumina NovaSeq 6000 (Novogene, Cambridge, United Kingdom) with 100 × coverage and 2 × 150 bp read length and a Phred score of Q30 ≥ 80%.

Raw reads have been deposited in the European Nucleotide Archive (ENA) at EMBL-EBI under accession number PRJEB47797. Accession numbers are provided in Supplementary Table 1.

### Bioinformatic Analyses

Bacterial DNA sequences were analyzed with Ridom SeqSphere+ software v7.0.4 (Ridom GmbH, Germany) (Jünemann et al., 2013) and Center for Genomic Epidemiology (CGE) web-based tools (DTU, Denmark) available at http://www.genomicepidemiology.org. Within Ridom SeqSphere+ pipeline, raw reads were assembled with SKESA v2.3.0 (Souvorov et al., 2018) together with quality control with FastQC v0.11.7 (Babraham Institute, 2021) and adapter trimming with Trimmomatic v0.36 (Bolger et al., 2014), AMR genes were identified with NCBI AMRFinderPlus v3.2.3 (Feldgarden et al., 2019), virulence genes with VFDB (Chen et al., 2016), and ST with *E. coli* MLST Warwick v1.0 (Achtman scheme) based on the PubMLST database (Jolley et al., 2018). Sequences from isolates with novel STs were submitted to the Enterobase database^1^ (Zhou et al., 2020) to assign new Achtman scheme STs.

Using default values, PlasmidFinder 2.0 (Carattoli et al., 2014) was employed to detect plasmid replicons and pMLST 2.0 (Carattoli et al., 2014) the plasmid multilocus ST for IncF and IncI1 type replicons. FimTyper 1.0 (Roer et al., 2017) was used to identify the *fimH* allele. ResFinder 4.1 (Camacho et al., 2009; Zankari et al., 2017; Bortolaia et al., 2020), SerotypeFinder 2.0 (Joensen et al., 2015), MLST 2.0 (Larsen et al., 2012), and VirulenceFinder 2.0 (Joensen et al., 2014; Tetzschner et al., 2020) were used to confirm acquired resistance genes, serotype, and ST, respectively.

#### Determination of C1-M27 Clade-Specific Prophage-Like Regions

Isolates were compared with BLASTn to strain KUN5781 (GenBank accession: LC209430) (Matsumura et al., 2016) to determine the presence of M27-C1 clade-specific prophage-like regions M27PP1 and M27PP2. Results were visualized with BRIG v0.95 (Alikhan et al., 2011) for isolates with matching regions.

#### Core Genome Multilocus Sequence Typing-Based Genetic Comparison

Core genome multilocus sequence typing (cgMLST) targeting 2520 genes was performed using Ridom SeqSphere+ software v7.0.4 (Ridom GmbH, Germany) (Jünemann et al., 2013) to compare all 30 isolates obtained from ISLAB and results were visualized with a minimum spanning tree (MST).

Isolates were also compared to available previously sequenced ESBL/AmpC-producing *E. coli* isolates from Finland (total *n* = 67) collected between 2012 and 2018 from broiler meat (*n* = 5) (Päivärinta et al., 2020), broiler caecum (*n* = 5) (Päivärinta et al., 2020), broiler production including broiler parents (*n* = 8) (Oikarainen et al., 2019), egg surfaces (*n* = 4) (Oikarainen et al., 2019) and production environment (*n* = 1) (Oikarainen et al., 2019), imported food products (*n* = 16) (Kurittu et al., 2021a), barnacle geese (*n* = 9) (Kurittu et al., 2021b), wastewater (*n* = 1) (unpublished), cattle (*n* = 1) (Päivärinta et al., 2016), veterinarians (*n* = 9) (Verkola et al., 2019), and healthy adults (*n* = 8) (Gröndahl-Yli-Hannuksela et al., 2020; Supplementary Table 2). cgMLST-based MST and was constructed with Ridom SeqSphere+ software.

## Results

### Extended-Spectrum Beta-Lactamase-Producing *Escherichia coli* From Clinical Samples

Altogether 30 *E. coli* isolates obtained from human clinical samples confirmed as ESBL-producing with phenotypic screening from the Eastern Finland healthcare district between 2018 and 2020 were subjected to WGS. Most isolates (*n* = 12; 40%) originated from a urine sample, followed by blood samples (*n* = 8; 27%). A majority of the isolates (*n* = 21; 70%) belonged to ST131, with the remaining nine isolates representing a different ST each, including ST38, ST1193, ST162, ST537, ST59, and ST405 (Table 1). A novel ST was identified in three isolates and the sequences were submitted to the Enterobase database (see text footnote 1) to assign new Achtman scheme STs. The newly assigned STs were as follows: ST12704, ST12703, and ST12705 for isolates D2, D4, and D9, respectively. Sequences from two isolates from previous studies, A41.2-1 and C76.1-2 (Kurittu et al., 2021a), were additionally submitted to Enterobase to assign new Achtman scheme STs. All ST131 isolates from the current study were of serotype O25:H4 and possessed the *fimH*30 allele, except for two isolates, which belonged to serotype O16:H5 and possessed the *fimH*41 allele.

**TABLE 1:** Genomic characterization of 30 ESBL-producing *Escherichia coli* isolates obtained from human clinical samples in the Eastern Finland healthcare district during 2018–2020.

Sample	Sample type	Sequence type	Serotype	*fimH* type	*bla* gene(s)	Plasmid replicon(s)	pMLST	Year of isolation
D1	Urine	ST131	O25:H4	*fimH*30	*bla*_*CTX–M–27*_	IncFIA, IncFIB, IncFIB(H89-PhagePlasmid), IncFII(pRSB107), IncX4	[F1:A2:B20]	2020
D2	Joint	ST12704	O4:H27	*fimH*2	*bla*_*CTX–M–15*_	IncFIA, IncFIB, IncFII(pRSB107)	[F1:A1:B10]	2018
D3	Scrotum	ST38	O1:H15	*fimH*65	*bla*_*CTX–M–27*_	IncFIA, IncFIB, IncFII(pRSB107), Col(BS512), Col156	[F1:A2:B20]	2018
D4	Maxillary sinus	ST12703	O18:H7*	*fimH*18	*bla*_*CTX–M–14*_, *bla*_*TEM–1*_	IncFIB	[F46:A-:B20]	2018
D5	Eye conjunctiva	ST1193	O75:H5	*fimH*64	*bla*_*CTX–M–55*_	IncB/O/K/Z, Col(BS512), Col(MG828)	–	2019
D6	Wound	ST131	O25:H4	*fimH*30	*bla*_*CTX–M–15*_, *bla*_*TEM–1*_	IncFIA, IncFIB, IncFII(pRSB107), Col156	[F1:A2:B20]	2019
D7	Blood	ST131	O25:H4	*fimH*30	*bla*_*CTX–M–15*_, *bla*_*OXA–1*_	No plasmid replicons found	–	2019
D8	Bile	ST131	O16:H5	*fimH*41	*bla*_*CTX–M–27*_	IncFIA, IncFIB, IncFII(pRSB107), IncX3, IncY, Col(BS512), Col156	[F1:A2:B20]	2019
D9	Abscess	ST12705	O16:H5	*fimH*41	*bla*_*CTX–M–15*_, *bla*_*TEM–1*_	IncFIB, IncFIB(H89-PhagePlasmid), IncFII(29), IncFII(pCoo)	[F29:A-:B10]	2019
D10	Blood	ST131	O25:H4	*fimH*30	*bla*_*CTX–M–15*_	IncFIA, IncFIB, Col(BS512)	[F36:A1:B20]**	2019
D11	Lung (bronchoalveolar lavage)	ST131	O25:H4	*fimH*30	*bla*_*CTX–M–27*_	IncFIA, IncFIB, IncFII(pRSB107), Col156	[F1:A2:B20]	2019
D12	Urine	ST131	O25:H4	*fimH*30	*bla*_*CTX–M–27*_	IncFIA, IncFIB, IncFII(pRSB107), IncI1	[F1:A2:B20]	2020
D13	Urine	ST131	O25:H4	*fimH*30	*bla*_*CTX–M–27*_	IncB/O/K/Z, IncFIA, IncFIB, IncFII, IncFII(pRSB107), Col156, Col8282	[F84:A2:B20]**	2019
D14	Blood	ST162	O8:H19	*fimH*32	*bla*_*SHV–12*_	IncFIA, IncFIC(FII), IncI1, IncQ1	[F18:A6:B-]**/ST26 CC-2	2019
D15	Urine	ST131	O25:H4	*fimH*30	*bla*_*CTX–M–15*_	IncFII, IncI1	[F2:A-:B-]/ST173	2019
D16	Urine	ST131	O25:H4	*fimH*30	*bla*_*CTX–M–27*_	IncFIA, IncFIB, IncFII(pRSB107), Col156	[F1:A2:B20]	2019
D17	Abdominal cavity	ST537	O75:H5	*fimH*5	*bla*_*TEM–52*_	IncI1	ST36/CC-3**	2019
D18	Urine	ST59	O1:H7	*fimH*41	*bla*_*CTX–M–55*_, *bla*_*TEM–1*_	IncFII(pCoo)	[F10:A-:B-]	2019
D19	Urine	ST405	O2:H4	*fimH*56	*bla*_*CTX–M–3*_	IncFIB, IncFII(29), IncI1, Col(BS512), Col156, Col156, Col156	[F29:A-:B10]/ST57 CC-5	2019
D20	Blood	ST131	O25:H4	*fimH*30	*bla*_*CTX–M–27*_	IncFIA, IncFIB, IncFII(pRSB107), Col156	[F1:A2:B20]	2020
D21	Blood	ST131	O25:H4	*fimH*30	*bla*_*CTX–M–27*_	IncFIA, IncFIB, IncFII(pRSB107)	[F1:A2:B20]**	2020
D22	Urine	ST131	O25:H4	*fimH*30	*bla*_*CTX–M–27*_	IncFIA, IncFIB, Col(pHAD28), Col156	[F-:A2:B20]	2020
D23	Wound	ST131	O25:H4	*fimH*30	*bla*_*CTX–M–15*_	IncFIA, IncFIB, Col(BS512)	[F-:A1:B20]**	2020
D24	Urine	ST131	O25:H4	*fimH*30	*bla*_*CTX–M–15*_, *bla*_*OXA–1*_	IncFIA, IncFIB, IncX4, Col156	[F-:A2:B20]	2020
D25	Blood	ST131	O25:H4	*fimH*30	*bla*_*CTX–M–27*_	IncFIA, IncFIB	[F-:A2:B20]	2020
D26	Urine	ST131	O25:H4	*fimH*30	*bla*_*CTX–M–27*_	IncFIA, IncFIB, IncFIB(H89-PhagePlasmid), IncFII(pRSB107), IncI1	[F1:A2:B20]/IncI1 unknown	2020
D27	Blood	ST131	O25:H4	*fimH*30	*bla*_*CTX–M–15*_	IncFIA, IncFIB, Col(BS512)	[F22:A1:B20]**	2020
D28	Blood	ST131	O16:H5	*fimH*41	*bla*_*CTX–M–15*_, *bla*_*TEM–1*_	IncFIB, IncFII(29), Col156	[F29:A-:B10]	2020
D29	Urine	ST131	O25:H4	*fimH*30	*bla*_*CTX–M–27*_	IncFIA, IncFIB, IncFII(pRSB107), Col156	[F1:A2:B20]	2020
D30	Urine	ST131	O25:H4	*fimH*30	*bla*_*CTX–M–27*_	IncFIA, IncFIB, Col(pHAD28), Col156	[F-:A2:B20]	2020

*SerotypeFinder 2.0 (Center for Genomic Epidemiology) used to verify result.

**Uncertain hit, ST cannot be trusted.

All isolates were found to match their respective phenotype genotypically, as all were genotypically confirmed to carry at least one *bla* gene representing an ESBL phenotype. The most common *bla* gene identified was *bla*_*CTX–M–27*_ (*n* = 14; 47%) followed by *bla*_*CTX–M–15*_ (*n* = 10; 33%). *bla*_*CTX–M–27*_ was identified in 13 out of the 21 isolates representing ST131 and additionally from one isolate of ST 38. Eight out of the 10 *bla*_*CTX–M–15*_ were harbored by ST131, with 7 representing the serotype O25:H4 together with *fimH* allele 30, while 1 was of serotype O16:H5 with *fimH*41 allele. Two isolates of novel STs, ST12704 (isolate D2) and ST12705 (isolate D9), were found to possess *bla*_*CTX–M–15*_. Regarding other *bla*_*CTX–M*_ genes, *bla*_*CTX–M–55*_ occurred in two isolates (D5 of ST1193 and D18 of ST59), and *bla*_*CTX–M–*14_ and *bla*_*CTX–M–3*_ each in one isolate each (D4 of ST12703 and D19 of ST405, respectively). *bla*_*SHV–12*_ was found from one isolate (D14) of ST 162 and *bla*_*TEM–52*_ from one isolate (D17) of ST 537.

All isolates were found to harbor at least one plasmid replicon, except for D7, from which no replicons were identified. Altogether 17 different replicons were detected with IncFIB (*n* = 24), IncFIA (*n* = 21), IncFII (*n* = 13), and Col156 (*n* = 13) type replicons were most prevalent. The majority of the isolates (*n* = 26; 87%) possessed multiple replicons, with IncF family plasmids appearing in 27 (90%) isolates. IncI1 plasmids were recovered from five isolates (D14, D15, D17, D19, and D26), all with varying pMLST profiles. Plasmids of pMLST IncF[F1:A2:B20] type were identified in 11 isolates, and pMLST IncF[F-:A2:B20] in 4 isolates.

Multidrug resistance, resistance to at least one agent in three or more antimicrobial categories (Magiorakos et al., 2012), was common among the isolates with multiple acquired resistance genes identified in 21 (70%) isolates, including genes against aminoglycosides, tetracycline, sulfonamides, macrolides, and trimethoprim (Table 2). Acquired sulfonamide resistance genes, *sul1* and *sul2*, were found either alone or together in 18 (60%) of the isolates.

**TABLE 2:** Virulence and antimicrobial resistance genes other than *bla* identified in 30 ESBL-producing *Escherichia coli* isolates obtained from Finnish patients collected in the Eastern Finland healthcare district during 2018–2020.

				Virulence factors				
Sample	Acquired resistance genes other than *bla*	Fosfomycin resistance mutations	Quinolone resistance mutations	Adherence	Invasion	Iron uptake	Toxins	Effector delivery system
D1	*aadA5, aph(3″)-Ib, aph(6)-Id, mph*(A), *sul1, sul2, tet*(A), *dfrA17*	*ptsI* (V25I), *uhpT* (E350Q)	*gyrA* (D87N), *gyrA* (S83L), *parC* (E84V), *parC* (S80I), *parE* (I529L)	*fimA, fimC, fimD, fimE, fimF, fimG, fimH, fimI, papB, papI, yagV/ecpE, yagW/ecpD, yagX/ecpC, yagY/ecpB, yagZ/ecpA, ykgK/ecpR*	*aslA, kpsD, kpsM, ompA*	*chuA, chuS, chuT, chuU, chuV, chuW, chuX, chuY, entB, entC, entE, entF, entS, fdeC, fepA, fepB, fepC, fepD, fepG, fes, iucA, iucB, iucC, iutA*	*sat*	
D2	*aph(3″)-Ib, aph(6)-Id, mph*(A), *sul2, dfrA14*	Not found	Not found	*fimA, fimB, fimC, fimD, fimE, fimF, fimG, fimH, fimI, yagV/ecpE, yagW/ecpD, yagX/ecpC, yagY/ecpB, yagZ/ecpA, ykgK/ecpR*	*aslA, ibeA, kpsD, kpsM, ompA*	*chuA, chuS, chuT, chuU, chuV, chuW, chuX, chuY, entB, entC, entD, entE, entS, fdeC, fepA, fepB, fepC, fepD, fepG, fes*	*pic, set1A, set1B, vat*	
D3	*aadA5, aph(3″)-Ib, aph(6)-Id, mph*(A), *sul1, sul2, tet*(A), *dfrA17*	Not found	*gyrA* (D87N), *gyrA* (S83L), *parC* (S80I)	*fdeC, fimA, fimB, fimC, fimD, fimE, fimF, fimG, fimH, fimI, papB, papI, papX, sfaX, yagV/ecpE, yagW/ecpD, yagX/ecpC, yagY/ecpB, yagZ/ecpA, ykgK/ecpR*	*aslA, kpsD, kpsM, ompA*	*chuA, chuS, chuT, chuU, chuV, chuW, chuX, chuY, entB, entC, entD, entE, entS, fepA, fepB, fepC, fepD, fepG, fes, iucA, iucB, iucC, iutA*	*sat*	*espL1, espL4, espR1, espX1, espX4, espX5, espY1, espY2, espY3, espY4*
D4	*tet*(M)	Not found	*marR* (S3N)	*fdeC, fimA, fimB, fimC, fimD, fimE, fimF, fimG, fimH, fimI, focC, focD, focF, focI, papB, papC, papD, papF, papI, papJ, papK, sfaA, sfaB, sfaC, sfaD, sfaE, sfaF, sfaG, sfaH, sfaS, sfaY, yagV/ecpE, yagW/ecpD, yagX/ecpC, yagY/ecpB, yagZ/ecpA, ykgK/ecpR*	*aslA, ibeA, kpsD, kpsM, kpsT, ompA*	*chuA, chuS, chuT, chuU, chuV, chuW, chuX, chuY, entB, entC, entD, entE, entS, fepA, fepB, fepC, fepD, fepG, fes, iroB, iroC, iroD, iroE, iroN*	*cnf1, hlyA, hlyB, hlyC, hlyD, vat*	
D5	Not found	*uhpT* (E350Q)	*gyrA* (D87N), *gyrA* (S83L), *marR* (S3N), *parC* (S80I), *parE* (L416F)	*fdeC, fimA, fimB, fimC, fimD, fimE, fimF, fimG, fimH, fimI, papB, papI, papX, sfaX, yagV/ecpE, yagW/ecpD, yagX/ec, yagY/ecpB, yagZ/ecpA, ykgK/ecpR*	*aslA, kpsD, kpsM, kpsT, ompA*	*chuA, chuS, chuT, chuU, chuV, chuW, chuX, chuY, entB, entC, entE, entS, fepA, fepB, fepC, fepD, fepG, fes, iucA, iucB, iucC, iutA*	*sat, vat*	
D6	*aadA5, aph(3″)-Ib, aph(6)-Id, mph*(A), *sul1, sul2, tet*(A), *dfrA17*	*ptsI* (V25I), *uhpT* (E350Q)	*gyrA* (D87N), *gyrA* (S83L), *parC* (E84V), *parC* (S80I), *parE* (I529L)	*fdeC, fimA, fimB, fimC, fimD, fimE, fimF, fimG, fimH, fimI, papB, papI, papX, sfaX, yagV/ecpE, yagW/ecpD, yagX/ecpC, yagY/ecpB, yagZ/ecpA, ykgK/ecpR*	*aslA, kpsM, ompA*	*chuA, chuS, chuT, chuU, chuV, chuW, chuX, chuY, entB, entC, entE, entF, entS, fepA, fepB, fepC, fepD, fepG, fes, iucA, iucB, iucC, iutA*	*sat*	
D7	*aac(6′)-Ib-cr*	*ptsI* (V25I), *uhpT* (E350Q)	*gyrA* (D87N), *gyrA* (S83L), *parC* (E84V), *parC* (S80I), *parE* (I529L)	*fdeC, fimA, fimC, fimD, fimE, fimF, fimG, fimH, fimI, papC, papD, papF, papG, papJ, papK, yagV/ecpE, yagW/ecpD, yagX/ecpC, yagY/ecpB, yagZ/ecpA, ykgK/ecpR*	*aslA, kpsD, kpsM, ompA*	*chuA, chuS, chuT, chuU, chuV, chuW, chuX, chuY, entB, entC, entE, entF, entS, fepA, fepB, fepC, fepD, fepG, fes, iucA, iucB, iucC, iutA*	*cnf1, hlyA, hlyB, hlyC, hlyD, sat*	
D8	*aadA5, aph(3″)-Ib, aph(6)-Id, mph*(A), *sul1, sul2, tet*(A), *dfrA17*	*ptsI* (V25I), *uhpT* (E350Q)	*gyrA* (D87N), *gyrA* (S83L), *parC* (E84V), *parC* (S80I), *parE* (I529L)	*fdec, fimA, fimB, fimC, fimD, fimE, fimF, fimG, fimH, fimI, papB, papI, papX, sfaX, yagV/ecpE, yagW/ecpD, yagX/ecpC, yagY/ecpB, yagZ/ecpA, ykgK/ecpR*	*aslA, kpsD, kpsM, ompA*	*chuA, chuS, chuT, chuU, chuV, chuW, chuX, chuY, entB, entC, entE, entS, fepA, fepB, fepC, fepD, fepG, fes, iucA, iucB, iucC, iutA*	*sat, vat*	
D9	Not found	*ptsI* (V25I), *uhpT* (E350Q)	*gyrA* (S83L), *parE* (I529L)	*fdeC, fimA, fimB, fimC, fimD, fimE, fimF, fimG, fimH, fimI, papB, papI, papX, sfaX, yagV/ecpE, yagW/ecpD, yagX/ecpC, yagY/ecpB, yagZ/ecpA, ykgK/ecpR*	*aslA, kpsD, kpsM, ompA*	*chuA, chuS, chuT, chuU, chuV, chuW, chuX, chuY, entB, entC, entE, entS, fepA, fepB, fepC, fepD, fepG, fes, iucA, iucB, iucC, iutA*	*sat*	
D10	*aadA2, mph*(A), *sul1, tet*(A), *dfrA12*	*ptsI* (V25I), *uhpT* (E350Q)	*gyrA* (D87N), *gyrA* (S83L), *parC* (E84V), *parC* (S80I), *parE* (I529L)	*fdeC, fimA, fimC, fimD, fimE, fimF, fimG, fimH, fimI, papC, papD, papF, papG, papJ, papK, yagV/ecpE, yagW/ecpD, yagX/ecpC, yagY/ecpB, yagZ/ecpA, ykgK/ecpR*	*aslA, kpsD, kpsM, ompA*	*chuA, chuS, chuT, chuU, chuV, chuW, chuX, chuY, entB, entC, entE, entF, entS, fepA, fepB, fepC, fepD, fepG, fes, iucA, iucB, iucC, iutA*	*hlyA, hlyB, hlyC, hlyD, sat*	
D11	*aadA5, aph(3″)-Ib, aph(6)-Id, mph*(A), *sul1, sul2, tet*(A), *dfrA17*	*ptsI* (V25I), *uhpT* (E350Q)	*gyrA* (D87N), *gyrA* (S83L), *parC* (E84V), *parC* (S80I), *parE* (I529L)	*fdeC, fimA, fimC, fimD, fimE, fimF, fimG, fimH, fimI, papB, papI, papX, sfaX, yagV/ecpE, yagW/ecpD, yagX/eC, yagY/ecpB, yagZ/ecpA, ykgK/ecpR*	*aslA, kpsD, kpsM, ompA*	*chuA, chuS, chuT, chuU, chuV, chuW, chuX, chuY, entB, entC, entE, entF, entS, fepA, fepB, fepC, fepD, fepG, fes, iucA, iucB, iucC, iutA*	*sat*	
D12	*aadA5, aph(3″)-Ib, aph(6)-Id, mph*(A), *sul1, sul2, tet*(A)	*ptsI* (V25I), *uhpT* (E350Q)	*gyrA* (D87N), *gyrA* (S83L), *parC* (E84V), *parC* (S80I), *parE* (I529L)	*fimA, fimC, fimD, fimE, fimF, fimG, fimH, fimI, papB, papI, yagV/ecpE, yagW/ecpD, yagX/eC, yagY/ecpB, yagZ/ecpA, ykgK/ecpR*	*aslA, kpsD, kpsM, ompA*	*chuA, chuS, chuT, chuU, chuV, chuW, chuX, chuY, entB, entC, entE, entF, entS, fepA, fepB, fepC, fepD, fepG, fes, iucA, iucB, iucC, iutA*	*sat*	
D13	Not found	Not found	*gyrA* (D87N), *gyrA* (S83L), *parC* (E84V), *parC* (S80I), *parE* (I529L)	*fdeC, fimA, fimC, fimD, fimE, fimF, fimG, fimH, fimI, papB, papX, sfaX, yagV/ecpE, yagW/ecpD, yagX/eC, yagY/ecpB, yagZ/ecpA, ykgK/ecpR*	*aslA, kpsD, kpsM, ompA*	*chuA, chuS, chuT, chuU, chuV, chuW, chuX, chuY, entB, entC, entE, entF, entS, fepA, fepB, fepC, fepD, fepG, fes, iucA, iucB, iucC, iutA*	*sat*	
D14	*aadA2, aph(3″)-Ib, aph(6)-Id, mph*(B), *cmlA1, sul1, sul2, tet*(A), *dfrA1*	Not found	*gyrA* (D87N), *gyrA* (S83L), *parC* (S80I)	*fdeC, fimA, fimB, fimC, fimD, fimE, fimF, fimG, fimH, fimI, papC, yagV/ecpE, yagW/ecpD, yagX/eC, yagY/ecpB, yagZ/ecpA, ykgK/ecpR*	*ompA*	*entB, entC, entD, entE, entS, fepA, febB, fepC, fepD, fepG, fes, iucA, iucB, iucC, iutA*	*astA, east1*	*espX1, espX4, espX5*
D15	Not found	*ptsI* (V25I), *uhpT* (E350Q)	*gyrA* (D87N), *gyrA* (S83L), *parC* (E84V), *parC* (S80I), *parE* (I529L)	*fdeC, fimA, fimC, fimD, fimE, fimF, fimG, fimH, fimI, papB, papI, papX, sfaX, yagV/ecpE, yagW/ecpD, yagX/eC, yagY/ecpB, yagZ/ecpA, ykgK/ecpR*	*aslA, kpsD, kpsM, ompA*	*chuA, chuS, chuT, chuU, chuV, chuW, chuX, chuY, entB, entC, entE, entF, entS, fepA, fepB, fepC, fepD, fepG, fes, iucA, iucB, iucC, iutA*	*sat*	
D16	*aadA5, aph(3″)-Ib, aph(6)-Id, mph*(A), *sul1, sul2, tet*(A), *dfrA17*	*ptsI* (V25I), *uhpT* (E350Q)	*gyrA* (D87N), *gyrA* (S83L), *parC* (E84V), *parC* (S80I), *parE* (I529L)	*fdeC, fimA, fimC, fimD, fimE, fimF, fimG, fimH, fimI, papB, papI, papX, sfaX, yagV/ecpE, yagW/ecpD, yagX/eC, yagY/ecpB, yagZ/ecpA, ykgK/ecpR*	*aslA, kpsD, kpsM, ompA*	*chuA, chuS, chuT, chuU, chuV, chuW, chuX, chuY, entB, entC, entE, entF, entS, fepA, fepB, fepC, fepD, fepG, fes, iucA, iucB, iucC, iutA*	*sat*	
D17	Not found	Not found	*marR* (S3N)	*fdeC, fimA, fimC, fimD, fimE, fimF, fimG, fimH, fimI, yagV/ecpE, yagW/ecpD, yagX/eC, yagY/ecpB, yagZ/ecpA, ykgK/ecpR*	*aslA, ibeA, kpsD, kpsM, ompA*	*chuA, chuS, chuT, chuU, chuV, chuW, chuX, chuY, entB, entC, entE, entS, fepA, fepB, fepC, fepD, fepG, fes*	*pic, set1A, set1B, vat*	
D18	Not found	*uhpT* (E350Q)	Not found	*fimA, fimB, fimC, fimD, fimE, fimF, fimG, fimH, fimI, papX, sfax*	*aslA, kpsD, kpsM, kpsT, ompA*	*chuA, chuS, chuT, chuU, chuV, chuW, chuX, chuY, entB, entC, entE, entS, fepA, fepB, fepC, fepD, fepG, fes, iucA, iucB, iucC, iucD, iutA*	*sat*	*espL1, espR1, espX1, espX4, espY2, espY4*
D19	Not found	Not found	Not found	*fdeC, fimA, fimB, fimC, fimD, fimE, fimF, fimG, fimH, fimI, papC, papD, papG, papI, papJ, papK, yagV/ecpE, yagW/ecpD, yagX/eC, yagZ/ecpA, ykgK/ecpR*	*aslA, kpsD, kpsM, ompA*	*chuA, chuS, chuT, chuU, chuV, chuW, chuX, chuY, entB, entC, entD, entE, entS, fepA, fepB, fepC, fepD, fepG, fes, iucA, iucB, iucC, iutA*	*hlyA, hlyB, hlyC, hlyD, sat*	*espL1, espL4, espX1, espX4, espX5, espY2, espY3, espY4*
D20	*aph(3″)-Ib, aph(6)-Id, sul2, tet*(A)	*ptsI* (V25I), *uhpT* (E350Q)	*gyrA* (D87N), *gyrA* (S83L), *parC* (E84V), *parC* (S80I), *parE* (I529L)	*fdeC, fimA, fimC, fimD, fimE, fimF, fimG, fimH, fimI, papB, papI, papX, sfax, yagV/ecpE, yagW/ecpD, yagX/ecpC, yagY/ecpB, yagZ/ecpA, ykgK/ecpR*	*aslA, kpsD, kpsM, ompA*	*chuA, chuS, chuT, chuU, chuV, chuW, chuX, chuY, entB, entC, entE, entF, entS, fepA, fepB, fepC, fepD, fepG, fes, iucA, iucB, iucC, iutA*	*sat*	
D21	Not found	*ptsI* (V25I), *uhpT* (E350Q)	*gyrA* (D87N), *gyrA* (S83L), *parC* (E84V), *parC* (S80I), *parE* (I529L)	*fdeC, fimA, fimC, fimD, fimE, fimF, fimG, fimH, fimI, papB, papI, papX, sfax, yagV/ecpE, yagW/ecpD, yagX/ecpC, yagY/ecpB, yagZ/ecpA, ykgK/ecpR*	*aslA, kpsD, kpsM*	*chuA, chuS, chuT, chuU, chuV, chuW, chuX, chuY, entB, entC, entE, entF, entS, fepA, fepB, fepC, fepD, fepG, fes, iucA, iucB, iucC, iutA*	*sat*	
D22	*aadA5, aph(3″)-Ib, aph(6)-Id, mph*(A), *sul1, sul2, tet*(A), *dfrA17*	*ptsI* (V25I), *uhpT* (E350Q)	*gyrA* (D87N), *gyrA* (S83L), *parC* (E84V), *parC* (S80I), *parE* (I529L)	*fdeC, fimA, fimC, fimD, fimE, fimF, fimG, fimH, fimI, papB, papI, yagV/ecpE, yagW/ecpD, yagX/ecpC, yagY/ecpB, yagZ/ecpA, ykgK/ecpR*	*aslA, kpsD, kpsM, ompA*	*chuA, chuS, chuT, chuU, chuV, chuW, chuX, chuY, entB, entC, entE, entF, entS, fepA, fepB, fepC, fepD, fepG, fes, iucA, iucB, iucC, iutA*	*sat*	
D23	*aadA2, mph(A), sul1, tet*(A), *dfrA12*	*ptsI* (V25I), *uhpT* (E350Q)	*gyrA* (D87N), *gyrA* (S83L), *parC* (E84V), *parC* (S80I), *parE* (I529L)	*fdeC, fimA, fimC, fimD, fimE, fimF, fimG, fimH, fimI, papC, papD, papF, papG, papJ, papK, yagV/ecpE, yagW/ecpD, yagX/ecpC, yagY/ecpB, yagZ/ecpA, ykgK/ecpR*	*aslA, kpsD, kpsM, ompA*	*chuA, chuS, chuT, chuU, chuV, chuW, chuX, chuY, entB, entC, entE, entF, entS, fepA, fepB, fepC, fepD, fepG, fes, iucA, iucB, iucC, iutA*	*hlyD, sat*	
D24	*aac(6′)-Ib-cr*	*ptsI* (V25I), *uhpT* (E350Q)	*gyrA* (D87N), *gyrA* (S83L), *parC* (E84V), *parC* (S80I), *parE* (I529L)	*fdeC, fimA, fimC, fimD, fimE, fimF, fimG, fimH, fimI, papB, papI, papX, sfaX, yagV/ecpE, yagW/ecpD, yagX/ecpC, yagY/ecpB, yagZ/ecpA, ykgK/ecpR*	*aslA, kpsD, kpsM, ompA*	*chuA, chuS, chuT, chuU, chuV, chuW, chuX, chuY, entB, entC, entE, entF, entS, fepA, fepB, fepC, fepD, fepG, fes, iucA, iucB, iucC, iutA*	*sat*	
D25	*aadA5, aph(3″)-Ib, aph(6)-Id, mph*(A), *sul1, sul2, tet*(A), *dfrA17*	*ptsI* (V25I), *uhpT* (E350Q)	*gyrA* (D87N), *gyrA* (S83L), *parC* (E84V), *parC* (S80I), *parE* (I529L)	*fdeC, fimA, fimC, fimD, fimE, fimF, fimG, fimH, fimI, papB, papI, papX, sfax, yagV/ecpE, yagW/ecpD, yagX/ecpC, yagY/ecpB, yagZ/ecpA, ykgK/ecpR*	*aslA, kpsD, kpsM, ompA*	*chuA, chuS, chuT, chuU, chuV, chuW, chuX, chuY, entB, entC, entE, entF, entS, fepA, fepB, fepC, fepD, fepG, fes, iucA, iucB, iucC, iutA*	*sat*	
D26	Not found	*ptsI* (V25I), *uhpT* (E350Q)	*gyrA* (D87N), *gyrA* (S83L), *parC* (E84V), *parC* (S80I), *parE* (I529L)	*fdeC, fimA, fimC, fimD, fimE, fimF, fimG, fimH, fimI, papB, papI, yagV/ecpE, yagW/ecpD, yagX/ecpC, yagY/ecpB, yagZ/ecpA, ykgK/ecpR*	*aslA, kpsD, kpsM, ompA*	*chuA, chuS, chuT, chuU, chuV, chuW, chuX, chuY, entB, entC, entE, entF, entS, fepA, fepB, fepC, fepD, fepG, fes, iucA, iucB, iucC, iutA*	*sat*	
D27	*aadA2, mph*(A), *sul1, tet*(A), *dfrA12*	*ptsI* (V25I), *uhpT* (E350Q)	*gyrA* (D87N), *gyrA* (S83L), *parC* (E84V), *parC* (S80I), *parE* (I529L)	*fdeC, fimA, fimC, fimD, fimE, fimF, fimG, fimH, fimI, papC, papD, papF, papG, papJ, papK, yagV/ecpE, yagW/ecpD, yagX/ecpC, yagY/ecpB, yagZ/ecpA, ykgK/ecpR*	*aslA, kpsD, kpsM, ompA*	*chuA, chuS, chuT, chuU, chuV, chuW, chuX, chuY, entB, entC, entE, entF, entS, fepA, fepB, fepC, fepD, fepG, fes, iucA, iucB, iucC, iutA*	*cnf1, hlyA, hlyB, hlyC, hlyD, sat*	
D28	*aadA5, aph(3″)-Ib, aph(6)-Id, mph*(A), *sul1, sul2, tet*(A), *dfrA17*	*ptsI* (V25I), *uhpT* (E350Q)	*gyrA* (S83L), *parE* (I529L)	*afaA, afaB-I, afaC-I, afaC-III, afaD, daaA, daaC, daaD, daaF, draA, draB, draC, draD, draP, fdeC, fimA, fimB, fimC, fimD, fimE, fimF, fimG, fimH, fimI, papB, papI, papX, sfaX, yagV/ecpE, yagW/ecpD, yagX/ecpC, yagY/ecpB, yagZ/ecpA, ykgK/ecpR*	*aslA, kpsD, kpsM, ompA*	*chuA, chuS, chuT, chuU, chuV, chuW, chuX, chuY, entB, entC, entE, entS, fepA, fepB, fepC, fepD, fepG, fes*	*hlyA, hlyB, hlyC, hlyD*	
D29	*aadA5, aph(3″)-Ib, aph(6)-Id, mph*(A), *sul1, sul2, tet*(A), *dfrA17*	*ptsI* (V25I), *uhpT* (E350Q)	*gyrA* (D87N), *gyrA* (S83L), *parC* (E84V), *parC* (S80I), *parE* (I529L)	*fdeC, fimA, fimC, fimD, fimE, fimF, fimG, fimH, fimI, papB, papI, papX, sfaX, yagV/ecpE, yagW/ecpD, yagX/ecpC, yagY/ecpB, yagZ/ecpA, ykgK/ecpR*	*aslA, kpsD, kpsM, ompA*	*chuA, chuS, chuT, chuU, chuV, chuW, chuX, chuY, entB, entC, entE, entF, entS, fepA, fepB, fepC, fepD, fepG, fes, iucA, iucB, iucC, iutA*	*sat*	
D30	*aadA5, aph(3″)-Ib, aph(6)-Id, mph*(A), *sul1, sul2, tet*(A), *dfrA17*	*ptsI* (V25I), *uhpT* (E350Q)	*gyrA* (D87N), *gyrA* (S83L), *parC* (E84V), *parC* (S80I), *parE* (I529L)	*fdeC, fimA, fimC, fimD, fimE, fimF, fimG, fimH, fimI, papB, papI, papX, sfaX, yagV/ecpE, yagW/ecpD, yagX/ecpC, yagY/ecpB, yagZ/ecpA, ykgK/ecpR*	*aslA, kpsD, kpsM, ompA*	*chuA, chuS, chuT, chuU, chuV, chuW, chuX, chuY, entB, entC, entE, entF, entS, fepA, fepB, fepC, fepD, fepG, fes, iucA, iucB, iucC, iutA*	*sat*	

Genes conferring trimethoprim resistance, either *dfrA17*, *dfrA12*, *dfrA1*, or *dfrA14*, were detected in 16 (53%) isolates. Of these genes, *dfrA17* was the most prevalent (*n* = 11; 37%), followed by *dfrA12* (*n* = 3; 10%). *dfrA1* and *dfrA14* appeared in one isolate each.

Aminoglycoside resistance [*aadA5*, *aadA2*, *aph(3″)-Ib*, *aph(6)-Id*, and/or *aac(6′)-Ib-cr*] was detected in 20 (67%) isolates and tetracycline resistance in 18 (60%) isolates [*tet*(A) in 17 and *tet*(M) in one isolate]. No carbapenemase genes were detected.

Chromosomal quinolone resistance mutations in *gyrA*, *parC*, *parE*, or *marR* were recovered from 27 of the 30 isolates, whereas plasmid-mediated quinolone resistance (PMQR) gene *aac(6′)-Ib-cr* was additionally identified in two isolates (D7 and D24). Chromosomal mutations in *ptsI* and *uhpt* associated with fosfomycin resistance were discovered in 23 (77%) isolates.

All the isolates harbored multiple virulence factors, with extraintestinal pathogenic *E. coli* (ExPEC) associated virulence genes (Johnson et al., 2003) *pap* (P fimbrial adhesin), *kpsMII* (polysialic acid transport protein; group 2 capsule), *iutA* (ferric aerobactin receptor), and *sfa* (S and F1C fimbriae) recovered from 29, 28, 26, and 18 isolates, respectively. Only isolate D2 lacked the previously described threshold of two or more of the five virulence genes (*pap*, *kps*, *iutA*, *sfa*/*foc*, *afa*/*dra*) defined as discriminatory for ExPEC classification (Johnson et al., 2003; Kanamori et al., 2017). No Shiga toxin (*stx*) genes were found. Virulence factors are presented according to their pathogenicity factor groups (Nesta et al., 2012; Pitout, 2012; Chen et al., 2016; Sarowska et al., 2019; Duan et al., 2020; Dekker et al., 2021) in Table 2 together with resistance genes other than *bla*.

Assembly statistics including the number of bases and contigs, the N50 value and average coverage for each isolate are available in Supplementary Table 3.

### Identification of C1-M27 Clade-Specific Prophage-Like Regions

**FIGURE 1:**
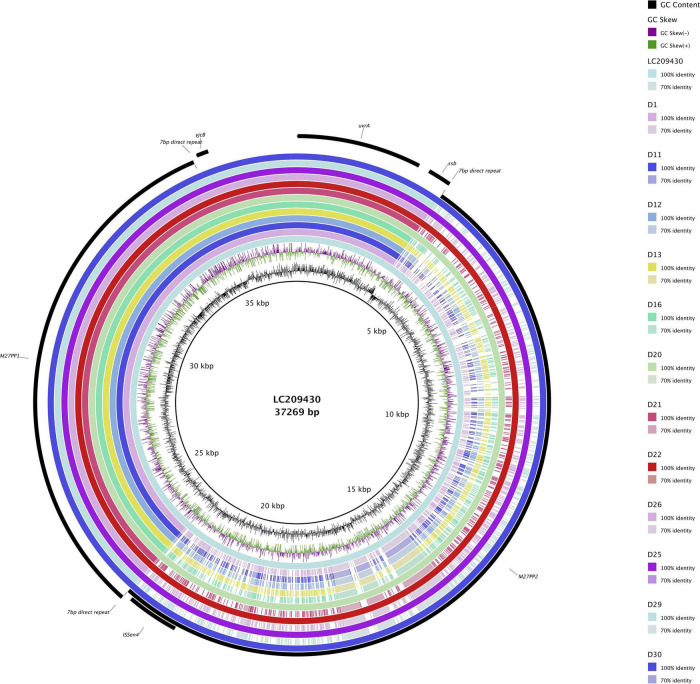
ST131 *Escherichia coli* C1-M27 clade-specific regions, prophage-like genomic islands M27PP1 and M27PP2, of KUN5781 (GenBank accession: LC209430) compared to CTX-M-27-producing ST131 *E. coli* with *fimH*30 allele from the current study. GC content and GC skew are depicted on the inner map with distance scale and predicted coding sequences depicted on the outer ring.

All 12 ST131 *E. coli* isolates with *bla*_*CTX–M–27*_ and *fimH*30 allele were found to possess the C1-M27 clade-specific prophage-like 11,894-bp region M27PP1 together with the 7 bp direct repeats (Matsumura et al., 2016; Figure 1). Four of these 12 samples additionally possessed the 19,352-bp prophage-like region M27PP2.

### Core Genome Multilocus Sequence Typing Comparison of Human Extended-Spectrum Beta-Lactamase-Producing *Escherichia coli* Isolates From the Eastern Finland Healthcare District

**FIGURE 2:**
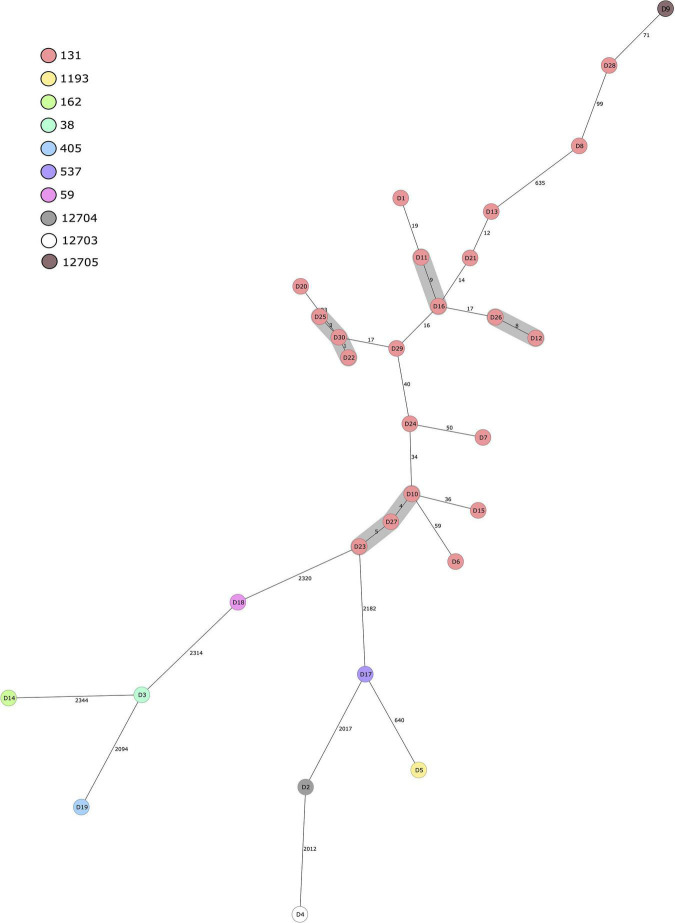
Minimum spanning tree of 30 human ESBL-producing *Escherichia coli* isolates obtained from patients in Eastern Finland during 2018–2020. Tree was calculated in Ridom SeqSphere+ with 2513 core genome multilocus sequence typing (cgMLST) targets and 7 *E. coli* MLST Warwick targets (pairwise ignoring missing values, logarithmic scale). Nodes are colored according to sequence type. Number of allelic differences between isolates are indicated on the connecting lines. Clusters are defined as ≤10 allelic difference and shaded in gray.

All of the 30 human ESBL-producing *E. coli* isolates obtained from the Eastern Finland healthcare district were compared with a cgMLST-based MST (Figure 2). Results indicate isolates within ST131 form clusters, meaning allelic differences were ≤10, whereas isolates of different STs are genetically distant with allelic differences ranging from 640 to over 2000 between two isolates. Three different clusters were observed within isolates belonging to the ST131 C1-M27 clade: D11 and D16, D26 and D12, and D25 together with D30 and D22. Additionally, one cluster between three isolates (D23, D27, and D10) was identified, comprising isolates with the same ST (ST131), serotype (O25:H4), fimH type (*fimH*30), and AMR gene resistance profile [*aadA2*, *bla*_*CTX–M–15*_, *mph*(A), *sul1*, *tet*(A), *dfrA12*].

### Core Genome Multilocus Sequence Typing Comparison to Previously Sequenced Extended-Spectrum Beta-Lactamase/AmpC-Producing *Escherichia coli* Isolates in Finland

**FIGURE 3:**
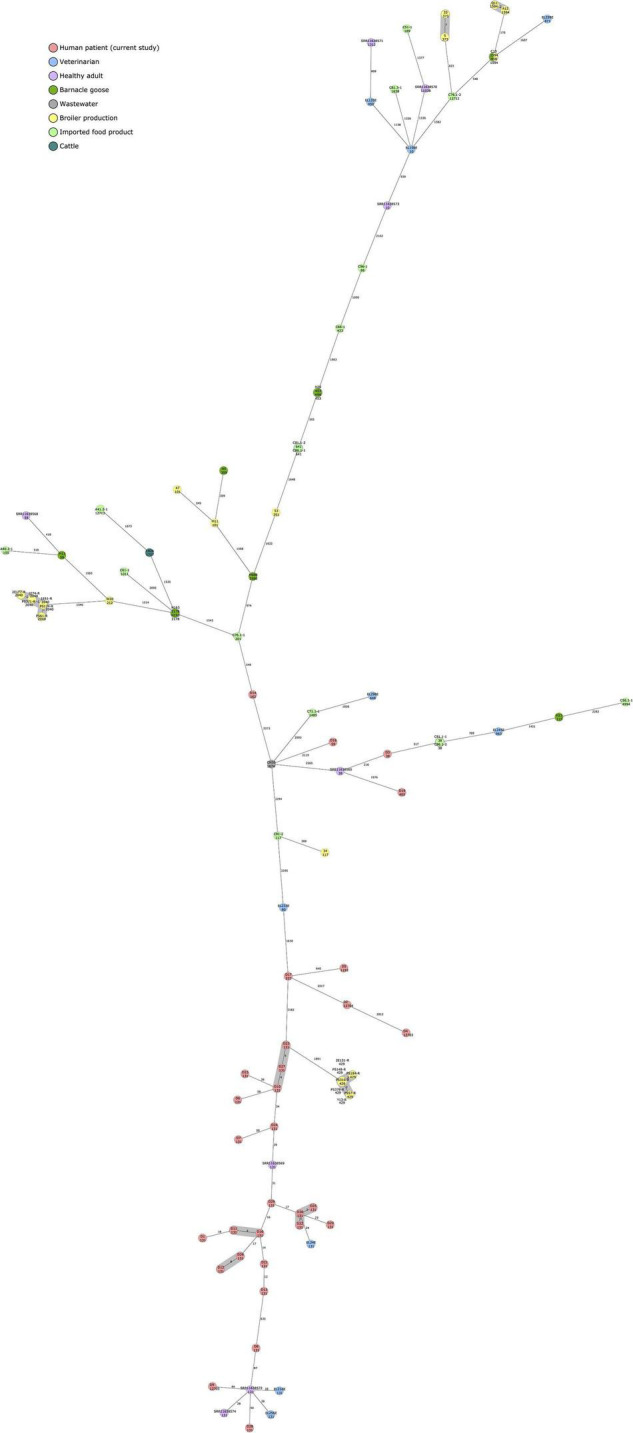
Minimum spanning tree of 97 ESBL/AmpC-producing *Escherichia coli* isolates calculated in Ridom SeqSphere+ with 2513 core genome multilocus sequence typing (cgMLST) targets and 7 *E. coli* MLST Warwick targets (pairwise ignoring missing values, logarithmic scale). Nodes are colored according to isolation source. Sequence type is indicated under the isolate name. Number of allelic differences between isolates are indicated on the connecting lines. Clusters are defined as ≤10 allelic difference and shaded in gray.

The 30 human ESBL-producing *E. coli* isolates obtained from the Eastern Finland healthcare district were additionally compared to available, previously sequenced ESBL/AmpC-producing *E. coli* isolates obtained from different sources in Finland (Figure 3). A cgMLST-based MST included 2520 gene targets.

Isolates obtained in the current study failed to form close clusters with isolates recovered from earlier studies, although the least distance (24 allelic difference) was observed between isolate D30 and EL24E, an isolate from a healthy veterinarian volunteer. Both isolates were of ST 131 and harbored *bla*_*CTX–M–27*_.

Other relatively close connections were also observed among isolates originating from human samples (18–44 allelic differences), all of ST 131, and positive for *bla*_*CTX–M–15*_. Two isolates from the current study, D9 and D28, differed by 44 and 40 alleles, respectively, from SRR11638572, an isolate recovered from a healthy Finnish volunteer. Two veterinarian isolates from a previously published study, EL216E and EL256E, differed by 18 and 26 alleles, respectively, from the same Finnish volunteer sample.

In addition to clusters observed within the isolates from the current study, isolates originating from poultry production (Oikarainen et al., 2019) formed two clusters with samples from the same study, and broiler meat (isolates 5 and 33) and broiler caecum (Q11 and A12) (Päivärinta et al., 2020) formed a cluster each. C15, a *bla*_*CMY–2*_-carrying ST1594 *E. coli* isolate from broiler caecum (Päivärinta et al., 2020), did not differ at all with cgMLST-based MST analysis from H58, an isolate originating from barnacle goose (Kurittu et al., 2021b).

## Discussion

We used WGS to characterize 30 ESBL-producing *E. coli* isolates obtained from clinical samples in Eastern Finland and performed cgMLST-based genomic comparisons to ESBL/AmpC-producing *E. coli* isolates of human, animal, food, and environmental origins isolated in Finland previously. ESBL-producing *E. coli* isolates from human sources were found to be genetically distinct from non-human sources in Finland. However, most ST131 *bla*_*CTX–M–27*_-positive *E. coli* isolates from human clinical samples were found to belong to a recently discovered international *E. coli* ST131 C1-M27 subclade, providing important insight to the epidemiology and increasing spread of this globally successful pathogenic clonal group. Strains within the C1-M27 clade carry *bla*_*CTX–M–27*_, possess *fimH* allele 30 and a prophage-like genomic island termed M27PP1, sometimes together with another prophage-like region, M27PP2 (Matsumura et al., 2016). *bla*_*CTX–M–27*_ has been noted to rival the globally dominant human-associated *bla*_*CTX–M–15*_ in many parts of the world, having been isolated from human, animal, food, or environmental sources in multiple countries in Europe, North America, and Asia (Bevan et al., 2017).

ST131 has become the dominant ExPEC lineage causing infections in humans worldwide (Banerjee and Johnson, 2014; Nicolas-Chanoine et al., 2014; Mathers et al., 2015). Subclones of ST131 *E. coli*, mainly H30 and H30Rx, are associated with fluoroquinolone resistance, and *bla*_*CTX–M–15*_ in the case of H30Rx (Banerjee et al., 2013). Previously *bla*_*CTX–M–15*_ has been the most prevalent *bla* gene identified from human isolates, but recent studies have noted a rise in the prevalence of *bla*_*CTX–M–27*_, starting from Japan in the late 2000s (Matsumura et al., 2016), and more recently a rapid increase in fecal carriage was observed in children in France (Birgy et al., 2017), along with human isolates from Germany (Ghosh et al., 2017), as well as from samples from hospitalized patients from four European cities (Berlin, Geneva, Madrid, and Utrecht) (Merino et al., 2018). Worldwide distribution is further demonstrated with the recent finding of ST131 *E. coli* belonging to C1-M27 clade in Brazil from a marine sample (Fernandes et al., 2020). Worryingly, ST131-*bla*_*CTX–M–27*_-*E. coli* has been noted to have a higher transmission rate compared to ST131-*bla*_*CTX–M–15*_-*E. coli* in an Israeli hospital setting (Adler et al., 2012). Our findings support the notion of a shift in the most dominant *bla*_*CTX–M*_ observed in human samples, and the emergence of *bla*_*CTX–M–27*_ as a challenger for *bla*_*CTX–M–15*_. Our findings regarding isolates within C1-M27 clade also are in line with previous studies where a majority of isolates were found to possess only the M27PP1 prophage-like region, instead of possessing both M27PP1 and M27PP2 (Matsumura et al., 2016; Decano and Downing, 2019). Interestingly, M27PP1 was also found with 100% coverage and 99.95% identity with BLASTn from sample D18, which carries *bla*_*CTX–M–55*_ and *bla*_*TEM–1*_ and is of ST 59 and of *fimH* type H41.

*bla*_*CTX–M–55*_ was identified in two of our isolates, D5 and D18, with different STs (ST1193 and ST59, respectively). This *bla* gene was additionally identified from two previously sequenced ESBL-producing *E. coli* isolates from Finland, one from an imported food sample (coriander from Malaysia) representing ST155 (Kurittu et al., 2021a) and one from a healthy, human adult fecal sample, representing ST58 (Gröndahl-Yli-Hannuksela et al., 2020). *bla*_*CTX–M–55*_-harboring *E. coli* has been reported especially in samples from meat and food-producing animals, as well as in humans in Asian countries (Zheng et al., 2012; Zhang et al., 2014; Zeng et al., 2021). Studies conducted in China have noted an increase in the proportion of *bla*_*CTX–M–55*_ compared to other prevalent *bla*_*CTX–M*_ genes, such as *bla*_*CTX–M–15*_ and *bla*_*CTX–M–14*_, in human patient material (Zhang et al., 2014; Zeng et al., 2021), depicting the rapidly evolving epidemiology of these enzymes. In addition to *bla*_*CTX–M–55*_, ST1193 *E. coli* was notably recognized as the most prevalent ST among uropathogenic *E. coli* (UPEC) isolates in female patients in China (Zeng et al., 2021). A rapid increase in ST1193 has also been detected in the United States from clinical fluoroquinolone-resistant *E. coli* isolates from urine samples (Tchesnokova et al., 2019). The only ST1193 isolate recovered in our study from an eye conjunctive sample (D5) harbored chromosomal quinolone resistance genes (*gyrA*, *marR*, *parC*, *parE*) in addition to *bla*_*CTX–M–55*_. Isolates from the previously mentioned study were, however, rarely resistant to beta-lactams, which was not the case in our sample. An Australian study has described fluoroquinolone-resistant ST131 and ST1193 *E. coli* as being less prevalent in animals compared to humans, and considers humans most likely as the source for possible findings of these bacteria in animals (Kidsley et al., 2020). Although not found in the comparative analysis of our study, ST131 *E. coli* isolates belonging to C1-M27 subclade have also been isolated previously from animal sources, more specifically from two pig isolates in the United Kingdom (Duggett et al., 2021) and companion animals in France (Melo et al., 2019).

A recent study in the United States found *bla*_*CTX–M–27*_ to be the second most common *bla* gene after *bla*_*CTX–M–15*_ in clinical human isolates, and notably *bla*_*CTX–M–27*_ was associated with ST38 *E. coli* (Mostafa et al., 2020). The *bla*_*CTX–M–27*_ gene on ST38 *E. coli* was found to be mostly plasmid-borne, residing in an IncF[F2:A-:B10] or IncF[F1:A2:B20] plasmid (Mostafa et al., 2020). One isolate (D3) in our study was positive for *bla*_*CTX–M–27*_-carrying ST38 *E. coli*, and this isolate harbored several IncF type replicons [IncFIA, IncFIB, IncFII(pRSB107)] together with Col plasmids [Col(BS512), Col156] and represented the replicon ST [F1:A2:B20].

The majority of our human clinical isolates harbored plasmid replicons belonging to the IncF family, which have been identified as important carriers of globally successful AMR genes, especially those encoding for ESBLs (Villa et al., 2010; Rozwandowicz et al., 2018). Thirteen of our isolates harbored an IncFII replicon together with FIA and FIB replicons, which together form a typical IncF multireplicon (Villa et al., 2010). The most common IncF replicon type identified in our isolates was [F1:A2:B20], which was found from 10 ST131 *E. coli* isolates carrying *bla*_*CTX–M–27*_ (*n* = 9) and *bla*_*CTX–M–15*_ (*n* = 1), and from one ST38 carrying *bla*_*CTX–M–27*_, similar to the findings of a study conducted in the United States (Mostafa et al., 2020). This supports the observation that plasmids belonging to pMLST [F1:A2:B20] are associated with the C1-M27 subclade (Ghosh et al., 2017; Mostafa et al., 2020). IncI1 plasmid replicons belonging to different pMLST profiles were identified in five isolates (D14, D15, D17, D19, and D26), together with *bla*_*SHV–12*_, *bla*_*CTX–M–15*_, *bla*_*TEM–52*_, *bla*_*CTX–M–3*_, and *bla*_*CTX–M–27*_, respectively. IncI type plasmids, especially with *bla*_*CTX–M–1*_, are frequently found from *E. coli* from poultry sources (Rozwandowicz et al., 2018).

The pMLST results should, however, be interpreted with care, since long-read sequencing would allow for more robust and accurate identification of plasmid structures and gene locations. As plasmids are important mediators of AMR worldwide (Carattoli, 2013; Rozwandowicz et al., 2018), further plasmid characterization through hybrid sequencing methods is warranted to investigate the epidemiological events in more detail in future studies. Another limitation of our study is the limited number of human clinical isolates analyzed, and the confined geographical origin of the samples. Our results do, however, represent a period of several years and multiple different specimen types, which provide an initial overview of the situation of ESBL-producing *E. coli* in clinical samples in Finland. Furthermore, our results strengthen the finding of *bla*_*CTX–M–27*_-harboring *E. coli* belonging to C1-M27 subclade gaining prevalence in Europe and describe the first published finding of C1-M27-clade isolates in Finland.

Multidrug resistance was common among the human clinical isolates analyzed in our study. Trimethoprim resistance is often associated with UPEC isolates, and our frequent finding of *dfrA17* and *dfrA12* genes is in line with a previous study conducted in Korea, which observed *dfrA17* and *dfrA12* as the most prevalent trimethoprim resistance genes in urinary tract isolates (Lee et al., 2001). Chromosomal quinolone resistance, as well as acquired tetracycline, aminoglycoside, and sulfonamide resistance genes, was also common in our human isolates.

Our findings are in line with earlier studies investigating the possible origins of ESBL-producing *E. coli* in humans. ESBL-producing *E. coli* isolates from human sources were found to be genetically distant from isolates obtained from food, animal, and environmental sources with a cgMLST-based MST approach. The total sample size, however, was relatively small, and closer genetic connections could possibly have been observed with a larger dataset. The only relatively close connections between human clinical isolates sequenced in this study and previously sequenced ESBL-producing *E. coli* isolates from Finland were observed among human-derived samples from veterinarians and a healthy, adult volunteer. The isolates represented ST131 and carried either *bla*_*CTX–M–27*_ or *bla*_*CTX–M–15*_, representing typical results for a human-derived sample. The only close connections among the other previously sequenced isolates from non-human sources in Finland were observed between isolates originating from poultry sources. Interestingly, ST1594 with AmpC type beta-lactamase, *bla*_*CMY–2*_, was identified from a broiler caecal sample and a barnacle goose fecal sample and showed no allelic variation in the cgMLST-based MST analysis.

A population-based modeling study with a larger dataset conducted in the Netherlands investigating the community-acquired ESBL-carriage and its attributable sources concluded that human-to-human transmission is the main route for acquiring ESBL *E. coli*, even though food, animals, and environmental sources were found to account for transmission to a lesser extent (Mughini-Gras et al., 2019). Another study conducted in Sweden found no evidence for clonal transmission events between ESBL/AmpC-producing *E. coli* in humans, animals, and the environment, but similarities were discovered in resistance genes and plasmids, indicating possible limited transmission potential (Börjesson et al., 2016).

In conclusion, *bla*_*CTX–M–27*_ was found to be the most prevalent ESBL gene in human clinical samples, and no clear evidence for animal, food, or environmental genetic overlap was observed in our dataset. Our results prove the spread of *E. coli* belonging to the C1-M27 clade has been successful, and WGS-based methods for surveillance of AMR trends and is effective and warranted for future studies. Surveillance studies are needed to detect the rapid evolution and epidemiology of ESBL genes, and future studies focusing on plasmid-mediated AMR spread are needed to assess the epidemiological links between different bacterial sources further.

## Data Availability Statement

The datasets presented in this study can be found in online repositories. The names of the repository/repositories and accession number(s) can be found in the article/Supplementary Material.

## Author Contributions

PK, AH, BK, and JJ contributed to the concept and design of the study. JK performed the sample collection. PK and BK analyzed the whole genome sequence data and performed the subsequent analysis and constructed images of the sequence analysis. PK drafted the manuscript. BK, AH, and JK revised the manuscript. All authors have read and approved the final draft of the manuscript.

## Conflict of Interest

The authors declare that the research was conducted in the absence of any commercial or financial relationships that could be construed as a potential conflict of interest. The reviewer MB declared a past collaboration with one of the authors, PK, to the handling editor.

## Publisher’s Note

All claims expressed in this article are solely those of the authors and do not necessarily represent those of their affiliated organizations, or those of the publisher, the editors and the reviewers. Any product that may be evaluated in this article, or claim that may be made by its manufacturer, is not guaranteed or endorsed by the publisher.

## Footnotes

1
https://enterobase.warwick.ac.uk/
